# Synthesis and Antimicrobial Evaluation of 5-Iodopyrimidine Analogs

**DOI:** 10.4103/0250-474X.59551

**Published:** 2009

**Authors:** N. M. Goudgaon, N. J. Basha, S. B. Patil

**Affiliations:** Department of Studies and Research in Chemistry, Gulbarga University, Gulbarga-585 106, India

**Keywords:** Antibacterial, antifungal, schiff bases, 5-Substituted pyrimidine

## Abstract

4-Substituted-5-iodo-2-benzylthiopyrimidines were prepared efficiently in three steps. 2-Benzylthiopyrimidine on iodination in presence of base gave 5-iodo-2-benzylthiopyrimidine (1), which on chlorination with excess of POCl_3_ furnished 4-chloro-5-iodo-2-benzylthiopyrimidine (2). Reaction of 2 with substituted aromatic amines, 2-aminopyridine and hydrazine hydrate yielded 4-amino-5-iodo-2-benzylthiopyrimidines 3(a-e), (3f) and (3g) respectively. Further, 4-hydrazino-5-iodo-2-benzylthiopyrimidine on condensation with substituted aromatic and heterocyclic aldehydes afforded the corresponding schiff bases 4(a-h). The structure of synthesized compounds have been established by spectral studies and elemental analysis. Synthesized compounds have been screened for antimicrobial activity. Compound 3f exhibited good antifungal activity against *A. niger*. The compounds 4a, 4c, 4d, 4g and 4h exhibited good antibacterial activity.

Pyrimidine derivatives have proved to be of great importance in exhibiting therapeutic applications[1]. A large number of pyrimidine nucleosides are clinically useful for the control of retroviral infections[2–5]. One of the important class of anti-herpetic nucleosides is series of 5-substituted uracil nucleosides such as (E)-5-(2-bromovinyl)-2'-deoxyuridine (BVdu)[6] showed specific antivaricella zoster virus (VZV) activity. In view of effect of 5-substitution on the activity of thiouracil, synthesis and antithyroid activity of several 5-substituted pyrimidine derivatives have been reported[7]. In addition, 5-alkyl or 5-aryl-substituted pyrimidine derivatives are useful intermediates in the synthesis of nucleosides[8]. Also, many 5-substituted pyrimidines have shown inhibitory activity against *Streptococcus faecalis* R growth[9] and some are evaluated as inhibitors of enzymes involved in the pyrimidine catabolism like dihydrouracil dehydrogenase and uridine phosphorylase[10]. Further, 5-iodo substituted pyrimidine analogs are known for their antimicrobial[11] and antiviral activity[12,13]. Synthesis and biological activities of several compounds derived from pyrimidine analogs were reported from our laboratory[10,14,15]. We report herein the synthesis and antimicrobial activities of 4-amino-5-iodo-2-benzylthiopyrimidines (3a-g) and 4-aryl/heteroarylidenehydrazino-5-iodo-2-benzylthiopyrimidines (4a-h).

Melting points were recorded by using Thomas-Hoover melting point apparatus and were uncorrected. IR spectra in KBr disc were recorded on Perkin-Elmer-Spectrum-one FT IR spectrophotometer (v_max_ in cm^−1^) and ^1^H NMR in DMSO-*d_6_* and/or CDCl_3_on amx 400, 400 MHz spectrophotometer using TMS as internal standard (chemical shift in δ ppm). Mass spectra were recorded on a Jeol SX 102 Mass spectrometer using argon/xenon (6kv, 10 mA) as the FAB gas. Purity of the compounds was checked by TLC using silica gel ‘G’ plates obtained from Whatman Inc, and a fluorescent indicator. 5-Iodo-2-benzylthiouracil (1) was prepared by following the known literature method[7].

General procedure for the synthesis of 4-chloro-5-iodo-2-benzylthiopyrimidine (2) is as follows, to a mixture of 5-iodo-2-benzylthiouracil (1) (0.01 mol) and POCl_3_ (0.06 mol) was refluxed for 1 h. Excess POCl_3_ was distilled under reduced pressure and the reaction mixture was poured into 200 ml of ice cold water. The solid separated was extracted with ether (3 × 100 ml) and the ether extracts were washed with 5% aq. NaHCO_3_ solution (3 × 100 ml), followed by water (3 × 100 ml). Ether layer was dried over anhydrous MgSO_4_. Solvent was evaporated to produce pale yellow syrup. The syrup slowly solidified and recrystallized from ethanol; 2: IR (KBr, cm^−1^): 3026 (aromatic C-H str), 1599 (C=N). ^1^H NMR (CDCl_3_): δ 8.69 (1H, s, Ar-H, Pyrimidine), 7.44-7.26 (5H, m, Ar-H), 4.36 (2H, s, S-CH_2_ -Ph).

General procedure for the synthesis of 4-amino-5-iodo-2-benzylthiopyrimidines (3a-g) is as follows, to a solution of 4-chloro-5-iodo-2-benzylthiopyrimidine (0.001 mol) in methanol (20 ml) and pyridine (0.5 ml), appropriate primary amine (0.001 mol) was added. The reaction mixture was refluxed for 4 h on a steam-bath. Excess of methanol was removed under reduced pressure and the residue triturated with a little crushed ice and aqueous layer was neutralized with 0.1N HCl. Solid separated was filtered and washed with cold water. Recrystallized the crude product from ethanol furnished the desired compounds (3a-g); 3a: IR (KBr, cm^−1^): 3379 (N-H), 1598 (C=N). ^1^H NMR (DMSO-d_6_): δ 8.27 (1H, s, Ar-H, pyrimidine), 7.68 (1H, s, N-H), 7.33-7.07 (9H, m, Ar-H), 4.17 (2H, s, S-CH_2_ -Ph), 2.27 (3H, s, CH_3_). MS m/z: 433 (M^+^) and fragmented peaks at 341, 308, 214. Analysis calculated for C_18_H_16_IN_3_S: C, 50.0; H, 3.73; N, 9.72. Found: C, 49.98; H, 3.70; N, 9.65%; 3b: IR (KBr, cm^−1^): 3300 (N-H), 1580 (C=N). ^1^H NMR (CDCl_3_): δ 8.22 (1H, s, Ar-H, pyrimidine), 8.03 (1H, s, N-H), 7.7-6.78 (9H, m, Ar-H), 4.31 (2H, s, S-CH_2_-Ph), 3.7 (3H, s, OCH_3_). Analysis calculated for C_18_H_16_IN_3_ OS: C, 48.21; H, 3.571; N, 9.372. Found: C, 48.18; H, 3.565; N, 9.365%; 3c: IR (KBr, cm^−1^): 3447 (N-H), 1603 (C=N), 1525 (NO_2_). ^1^H NMR (CDCl_3_): δ 8.5 (1H, s, Ar-H, pyrimidine), 7.4-7.26 (10H, m, Ar-H, N-H), 4.38 (2H, s, S-CH_2_-Ph). Analysis calculated for C_17_H_13_IN_4_O_2_S: C, 43.83; H, 2.281; N, 12.09. Found: C, 43.78; H, 2.275; N, 12.05%; 3d: IR (KBr, cm^−1^): 3356 (N-H), 1602 (C=N), 1539 (NO_2_). ^1^H NMR (DMSO-d_6_): δ 8.69 (1H, s, Ar-H, pyrimidine), 8.4 (1H, s, N-H) 7.98-7.22 (9H, m, Ar-H), 4.39 (2H, s, S-CH_2_-Ph); 3e: IR (KBr, cm^−1^): 3402 (NH_2_), 3303 (NH), 1616 (C=N). ^1^H NMR (CDCl_3_): δ 8.34 (1H, s, Ar-H, pyrimidine), 7.23-6.8 (12H, m, Ar-H, N-H), 4.12 (2H, s, S-CH_2_-Ph). MS m/z: 434 (M^+^) and fragmented ion peaks at 342, 214. Analysis calculated for C_17_H_15_IN_4_S: C, 47.11; H, 3.464; N, 12.93. Found: C, 47.08; H, 3.460; N, 12.88%; 3f: IR (KBr, cm^−1^): 3427 (N-H), 1602 (C=N). ^1^H NMR (CDCl_3_)_:_ δ 8.5 (1H, s, Ar-H, pyrimidine), 7.40-7.26 (10H, m, Ar-H, N-H), 4.38 (2H, s, S-CH_2_-Ph). Analysis calculated for C_16_H_13_IN_4_S: C, 45.73; H, 3.126; N, 13.33. Found: C, 45.71; H, 3.120; N, 13.28%; 3g: IR (KBr, cm^−1^): 3303 (NH_2_), 3239 (N-H), 1624 (C=N). ^1^H NMR (DMSO-d_6_): δ 7.82 (1H, s, Ar-H, pyrimidine), 7.29 (1H, s, N-H), 7.26-6.9 (5H, m, Ar-H), 4.04 (2H, s, S-CH_2_-Ph), 3.83 (2H, s, NH_2_). MS m/z: 358 (M^+^) and fragmented ion peak at 342, 231. Analysis calculated for C_11_H_11_IN_4_S: C, 36.97; H, 3.083; N, 15.68. Found: C, 36.90; H, 3.080; N, 15.64%.

General procedure for the synthesis of 4-aryl/heteroarylidinehydrazino-5-iodo-2-benzylthiopyrimidines (4a-h) as follows, to a solution of 4-hydrazino-5-iodo-2-benzylthiopyrimidine (3 g, 0.001 mol) in ethanol (20 ml) and catalytic amount of concentrated HCl, appropriate aromatic aldehyde (0.001 mol) was added. The reaction mixture was refluxed for 4 h. Excess ethanol was removed under reduced pressure, solid separated was filtrated and recrystalised from ethanol to get the desired compounds; 4a: IR (KBr, cm^−1^): 3419 (N-H), 1628 (C=N). ^1^H NMR (DMSO-d_6_): δ 8.69 (1H, s, Ar-H, pyrimidine), 8.27 (1H, s, N-H), 7.62 (1H, s, N=CH), 7.37-6.84 (10H, m, Ar-H, O-H), 4.41 (2H, s, S-CH_2_-Ph). Mass: m/z 462 (M^+^) and fragmented ion peak at 385, 336. Analysis calculated for C_18_H_15_IN_4_OS: C, 46.76; H, 3.271; N, 12.13. Found: C, 46.70; H, 3.270; N, 12.08%; 4b: IR (KBr, cm^−1^): 3414 (N-H), 1608 (C=N). ^1^H NMR (DMSO-d_6_): δ 8.42 (1H, s, Ar-H, pyrimidine), 8.28 (1H, s, NH), 7.72 (1H, s, N=CH), 7.69-6.83 (9H, m, Ar-H), 4.39 (2H, s, S-CH_2_-Ph), 3.7 (3H, s, OCH_3_). MS m/z: 476 (M^+^) and fragmented ion peak at 455, 349. Analysis calculated for C_19_H_17_IN_4_OS: C, 48.01; H, 3.57; N, 11.78. Found: C, 47.98; H, 3.5; N, 11.77%; 4c: IR (KBr, cm^−1^): 3380 (N-H), 1616 (C=N). ^1^H NMR (DMSO-d_6_): δ 8.32 (1H, s, Ar-H, pyrimidine), 8.25 (1H, s, N-H), 7.54 (1H, s, N=CH), 7.5-6.8 (9H, m, Ar-H, OH), 4.42 (2H, s, S-CH_2_-Ph), 3.59 (3H, s, OCH_3_). Analysis calculated for C_19_H_17_IN_4_O_2_S: C, 46.43; H, 3.462; N, 11.38. Found: C, 46.39; H, 3.460; N, 11.37%; 4d: IR (KBr, cm^−1^): 3453 (N-H), 1604 (C=N). ^1^H NMR (DMSO-d_6_): δ 8.49 (1H, s, Ar-H, pyrimidine), 8.3 (1H, s, N-H), 7.68-7.14 (10H, m, Ar-H, N=CH), 4.41 (2H, s, S-CH_2_-Ph). MS: m/z 480 (M^+^) and fragmented ion peak at 369, 353; 4e: IR (KBr, cm^−1^): 3400 (N-H), 1618 (C=N); ^1^H NMR (DMSO-d_6_): δ 8.73 (1H, s, Ar-H, pyrimidine), 8.04 (1H, s, N-H), 7.26 (1H, s, N=CH), 7.19-6.87 (9H, m, Ar-H), 4.16 (2H, s, S-CH_2_-Ph). Analysis calculated for C_18_H_14_ClIN_4_S: C, 45.04; H, 2.919; N, 11.67. Found: C, 45.01; H, 2.915; N, 11.65%; 4f: IR (KBr, cm^−1^): 3440 (N-H), 1616 (C=N). ^1^H NMR (DMSO-d_6_): δ 8.76 (1H, s, Ar-H, pyrimidine), 8.66 (1H, s, N-H), 8.36 (1H, s, N=CH), 8.05-7.04 (8H, m, Ar-H), 4.41 (2H, s, S-CH_2_-Ph). Analysis calculated for C_16_H_13_IN_4_S_2_: C, 42.57; H, 2.882; N, 12.41. Found: C, 42.54; H, 2.886; N, 12.42%; 4g: IR (KBr, cm^−1^): 3356 NH_2,_ 1605 C=N. ^1^H NMR (DMSO-d_6_): δ 9.2 (1H, s, Ar-H, pyrimidine), 8.66 (1H, s, N-H), 8.39 (1H, s, N=CH), 8.39-6.82 (10H, m, Ar-H, NH_2_), 4.43 (2H, s, S-CH_2_-Ph). MS: m/z 462 (M^+^) and fragmented ion peak at 325, 275. Analysis calculated for C_17_H_15_IN_6_S: C, 44.27; H, 3.253; N, 18.22. Found: C, 44.24; H, 3.246; N, 18.20%; 4h: IR (KBr, cm^−1^): 3380 (N-H), 1616 (C=N); ^1^H NMR (DMSO-d_6_): δ 9.1 (s, 1H, Ar-H, pyrimidine), 9.01 (1H, s, N-H), 7.85-7.42 (17H, m, Ar-H, N=CH), 4.43 (2H, s, S-CH_2_-Ph). MS m/z: 588 (M^+^) and fragmented ion peak at 493, 295. Analysis calculated for C_27_H_21_IN_6_S_2_: C, 55.19; H, 3.577; N, 14.31. Found: C, 55.12; H, 3.569; N, 14.25%.

The antimicrobial activities were performed by cup plate method[14]. The sample was dissolved in DMF at the concentration of 1000 μg/ml. Compounds were screened for antibacterial activity against *P. aeruginosa, E. coli, B. subtilis* and *S. aureus*. Antifungal activity was carried out against *A. terrus* and *A. niger* under aseptic conditions. Gentamycine and fluconazole were used as standard drug for antibacterial and antifungal activities, respectively. The zone of inhibition was compared with standard drug after 24 h of incubation at 25° for antibacterial activity and 48 h at 30° for antifungal activity.

The starting material 4-chloro-5-iodo2-benzylthiopyrimidine (2) was prepared by chlorination of 5-iodo-2-benzylthiouracil with excess POCl_3_ (Scheme 1). Compound 2 obtained as yellow crystals having the melting point 90-91° in 80% yield. IR spectrum of compound 2 exhibited absorption at 3026 and 1599 cm^−1^ due to (aromatic C-H str), (C=N), respectively. ^1^H NMR signals are at δ 8.69 (1H, s, Ar-H, pyrimidine), 7.44-7.26 (5H, m, Ar-H), 4.36 (2H, s, S-CH_2_-Ph). Reaction of 2 with various substituted aromatic amines, 2-aminopyridine and hydrazine hydrate gave desired compounds 3a-g in 40-75% yield. Compound 3a obtained as yellow colored solid having melting point 80-82° in 55% yield. IR spectrum of compound 3a shows characteristic absorption of (N-H) at 3379, (C=N) at 1598 cm^−1^. ^1^H NMR signals at δ 8.27 (1H, s, Ar-H, pyrimidine), 7.68 (1H, s, N-H), 7.33-7.07 (9H, m, Ar-H), 4.17 (2H, s, S-CH_2_-Ph), 2.27 (3H, s, CH_3_). Further compound 3a was confirmed by mass spectra analysis, the molecular ion peak at 433 (M^+^) and fragmented ion peaks at 342, 214. Compound 3g obtained as colorless crystals having the melting point 120-121° in 65% yield. IR spectrum of compound 3g shows absorption of (NH_2_) at 3303, (N-H) at 3239 and (C=N) at 1624 cm^−1^. The ^1^H NMR of 3g shows signal at δ 7.82 (1H, s, Ar-H, pyrimidine), 7.29 (1H, s, NH), 7.26-6.9 (5H, m, Ar-H), 4.04 (2H, s, S-CH_2_-Ph), 3.83 (2H, s, NH_2_). Mass spectra of compound 3g shows molecular ion peak at 358 (M^+^) and fragmented ion peak at 342, 231. Reaction of compound 3g with substituted aromatic and heterocyclic aldehydes furnished desired compounds 4-(aryl/heteroarylidinehydrazino)-5-iodo-2-benzylthiopyrimidines (4a-h) in 60-75% yield. The compound 4a was obtained as yellow colored solid in 75% yield, having m.p. 210-212°. The IR spectrum of compound 4a shows characteristic absorption of (O-H) at 3419, (N-H) at 3166 and (C=N) at 1621 cm^−1^. The ^1^H-NMR signals are at δ 8.6 (1H, s, Ar-H, pyrimidine), 8.2 (1H, s, NH), 7.6 (1H, s, N=CH), 7.3-6.8 (10H, m, Ar-H, OH).,4.4 (2H, s, S-CH2-Ph). Further compound 4a was confirmed by mass spectral analysis, the molecular ion peak at 462 (M^+^) and fragmented ion peak at 385, 336. Compound 4h was obtained as green colored solid in 52% yield, having m.p.210-211°. IR spectrum of compound 4h shows characteristic absorption of (N-H) at 3380, (C=N) at 1616 cm^−1^. ^1^H NMR signals are at δ 9.1 (1H, s, Ar-H, pyrimidine), 9.01 (1H, s, NH), 7.85-7.42 (17H, m, Ar-H, N = CH), 4.43 (2H, s, S-CH_2_-Ph). Further compound 4h was confirmed by mass spectra analysis, the molecular ion peak at 588 (M^+^), fragmented ion peak at 495, 293. The physical constant of all the compounds are given in Table 1.

**Scheme 1:**
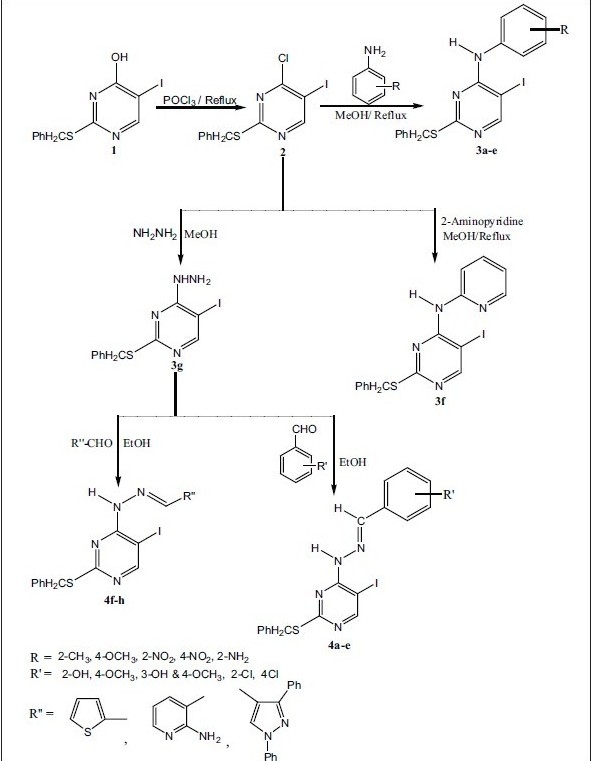
Synthesis of 5-substituted pyrimidine analogs

**TABLE 1:** PHYSICAL CONSTANTS OF THE SYNTHESIZED COMPOUNDS

Compd	R	R'	R″	Yield (%)	M.P(°)
3a	4-CH_3_	-	-	55	80-82
3b	4-OCH_3_	-	-	50	152-154
3c	2-NO_2_	-	-	55	60-62
3d	4-NO_2_	-	-	40	120-121
3e	2-NH_2_	-	-	50	160-161
3f	-	-	-	45	Semi-solid
3g	-	-	-	65	120-121
4a	-	2-OH	-	75	170-172
4b	-	4-OCH_3_	-	75	202-203
4c	-	3-OH, 4-OCH_3_	-	70	190-191
4d	-	4-Cl	-	75	210-213
4e	-	2-Cl	-	70	182-183
4f	-	-	Thiophene-2-yl	65	206-208
4g	-	-	2-Aminopyridin-3-yl	60	202-203
4h	-	-	1,3- Diphenyl-pyrazol-4-yl	52	210-211

All the compounds showed poor activity against gram (−)ve bacteria *E. coli* (Table 2). However compound 2 exhibited good antibacterial activity against gram (−)ve bacteria *P. aeruginosa* and good antifungal activity against both fungal strains *A. niger* and *A. terrus*. Compound 3d exhibited good antibacterial activity against gram (+)ve bacteria *S. aureus*. Compounds 3f exhibited good activity against *A. niger*. Compounds 4a, 4c and 4d showed good activity against *P. aeruginosa*. Compounds 4g and 4h exhibited good antibacterial activity against *P. aeruginosa, S. aureus* and *B. subtilis* and antifungal activity against both fungal strains. Other compounds have shown poor to moderate antibacterial and antifungal activity as compared to standard gentamycine and fluconazole. From the above results, it can be concluded that introduction of NO_2_, OH, Cl and heterocyclic moieties like pyridine and pyrazole to the pyrimidine analogs enhanced the antibacterial and antifungal activities.

**TABLE 2:** ANTIMICROBIAL ACTIVITIES OF SYNTHESIZED COMPOUNDS

Compd No	Dose (μg/ml)	Antibacterial Activity				Antifungal Activity	
			
		Zone of Inhibition* (mm)				Zone of Inhibition* (mm)	
			
		*P. aeruginosa*	*E. coli*	*B. subtilis*	*S. aureus*	*A. niger*	*A. terrus*
2	1000	19	05	15	10	12	11
3a	1000	07	09	10	11	17	17
3b	1000	06	08	12	13	10	10
3c	1000	07	10	10	11	12	11
3d	1000	10	05	14	16	15	13
3e	1000	08	15	11	14	13	12
3f	1000	05	10	09	15	19	14
3g	1000	19	04	10	11	10	10
4a	1000	18	07	13	15	13	12
4b	1000	15	05	12	12	11	15
4c	1000	19	04	13	12	12	11
4d	1000	18	08	10	10	10	12
4e	1000	19	06	08	09	14	11
4f	1000	10	05	15	12	15	10
4g	1000	18	07	17	18	18	15
4h	1000	16	10	20	18	16	15
Control (DMF)	-	Nil	Nil	Nil	Nil	Nil	Nil
Gentamycine	1000	22	19	24	22	-	-
Flucanazole	1000	-	-	-	-	22	20

* Zone of inhibition excluding well size 6 mm

## References

[1] Jain KS, Chitre TS, Maniyar PB, Kathiravan MK, Bindre VS, Veer VS (2006). Biological and medicinal significance of pyrimidine. Current Sci.

[2] Mitsuya H, Weinhold KJ, Furman PA, St.Clair MH, Nusinoff-Lehman S, Gallo RC (1985). 3'-Azido-3'-deoxythymidine (BW A509U): An antiviral agent that inhibits the infectivity and cytopathic effect of human T-lymphotropic virus type III/lymphadenopathy-associated virus in vitro. Proc Natl Acad Sci USA.

[3] Lin TS, Guo JY, Schinazi RF, Chu CK, Xiang JN, Prusoff WH (1988). Synthesis and antiviral activity of various 3-azido analogues of pyrimidine deoxyribonucleosides against human immunodeficiency virus (HIV-1, HTLV-III/LAV). J Med Chem.

[4] Choo H, Chong Y, Choi Y, Mathew J, Schinazi RF, Chu CK (2003). Synthesis, anti-HIV activity and molecular mechanism of drug resistance of L-2'.3'-didehydro-2',3'-dideoxy-2'-fluoro-4'-thionucleosides. J Med Chem.

[5] Balzarini J, Pannecouque C, De Clercq E, Aquaro S, Perno CF, Egberink H (2002). Antiretrovirus activity of a novel class of acyclic pyrimidine nucleoside phosphonates. Antimicrob Agents Chemother.

[6] De Clerecq E, Descamps J, De Somer P, Barr PJ, Jones AS, Walker RT (1979). (E)-5-(2-bromovinyl)-2-deoxyuridine: a potent and selective anti-herpes agents. Prot Natl Acad Sci USA.

[7] Barrett HW, Goodman I, Dittmer K (1948). The synthesis of 5-halogeno-2-thiouracil and 6-methyl-5-halogeno-2-thiouracil derivatives. J Am Chem Soc.

[8] Schinazi RF, Arbiser J, Lee J, Kalman T, Prusoff W (1986). Synthesis and biological activity of 5-phenylselenenyl-substituted pyrimidine. J Med Chem.

[9] Puleston HS, Charles FP, Norman FW (1955). Inhibition studies with pyrimidines on *Streptococcus faecalis* R. J Biol Chem.

[10] Goudgaon NM, Naguib FNM, El-Kouni MH, Schinazi RF (1993). Phenylselenenyl and phenylthio-substituted pyrimidines as inhibitors of dihydrouracil dehydrogenase and uridine phosphorylase. J Med Chem.

[11] Melvin DT, Anthony BN, Kyoichi AW, Jack JF (1981). Evaluation of the antiherpetic activity of 2-fluoro-5-iodo-Ara-c in rabbit eyes and cell cultures. Invest Opthlmol Vis Sci.

[12] Kaufman HE, Martola EL, Dolman H (1962). Use of 5-iodo-2'-deoxyuridine in the treatment of herpes simplex keratitis. Arch Opthalmol.

[13] Kuafman HE, Nesburn AB, Maloney ED (1962). IDU therapy of experimental herpes simplex keratitis. Arch Opthalmol.

[14] Goudgaon NM, Macmillan A, Schinazi RF (1992). 1-(Ethoxymethyl)-6-(phenylselenenyl)pyrimidines with activity against human immunodeficiency viruses types 1 and 2. Antiviral Chem Chemother.

[15] Goudgaon NM, Vijayalaxmi A (2003). Antimicrobial activity and structure-activity relationship of acyclic nucleosides. Indian J Pharm Sci.

